# X-treme loss of sequence diversity linked to neo-X chromosomes in filarial nematodes

**DOI:** 10.1371/journal.pntd.0009838

**Published:** 2021-10-27

**Authors:** John Mattick, Silvia Libro, Robin Bromley, Wanpen Chaicumpa, Matthew Chung, Darren Cook, Mohammad Behram Khan, Nikhil Kumar, Yee-Ling Lau, Shailja Misra-Bhattacharya, Ramakrishna Rao, Lisa Sadzewicz, Atiporn Saeung, Mohd Shahab, Benjamin C. Sparklin, Andrew Steven, Joseph D. Turner, Luke J. Tallon, Mark J. Taylor, Andrew R. Moorhead, Michelle Michalski, Jeremy M. Foster, Julie C. Dunning Hotopp

**Affiliations:** 1 Institute for Genome Science, University of Maryland, Baltimore, Maryland, United States of America; 2 New England Biolabs, Ipswich, Massachusetts, United States of America; 3 Department of Parasitology, Faculty of Medicine Siriraj Hospital, Mahidol University, Bangkok, Thailand; 4 Centre for Neglected Tropical Diseases, Department of Tropical Disease Biology, Liverpool School of Tropical Medicine, Liverpool, United Kingdom; 5 Department of Parasitology, Faculty of Medicine, University of Malaya, Kuala Lumpur, Malaysia; 6 Division of Parasitology, CSIR-Central Drug Research Institute, Lucknow, India; 7 Division of Infectious Diseases, Washington University School of Medicine, St Louis, Missouri, United States of America; 8 Center of Insect Vector Study, Department of Parasitology, Faculty of Medicine, Chiang Mai University, Chiang Mai, Thailand; 9 Department of Infectious Diseases, College of Veterinary Medicine, University of Georgia, Athens, Georgia, United States of America; 10 University of Wisconsin Oshkosh, Oshkosh, Wisconsin, United States of America; 11 Department of Microbiology and Immunology, University of Maryland, Baltimore, Maryland, United States of America; 12 Greenebaum Cancer Center, University of Maryland, Baltimore, Maryland, United States of America; Washington University School of Medicine, UNITED STATES

## Abstract

The sequence diversity of natural and laboratory populations of *Brugia pahangi* and *Brugia malayi* was assessed with Illumina resequencing followed by mapping in order to identify single nucleotide variants and insertions/deletions. In natural and laboratory *Brugia* populations, there is a lack of sequence diversity on chromosome X relative to the autosomes (π_X_/π_A_ = 0.2), which is lower than the expected (π_X_/π_A_ = 0.75). A reduction in diversity is also observed in other filarial nematodes with neo-X chromosome fusions in the genera *Onchocerca* and *Wuchereria*, but not those without neo-X chromosome fusions in the genera *Loa* and *Dirofilaria*. In the species with neo-X chromosome fusions, chromosome X is abnormally large, containing a third of the genetic material such that a sizable portion of the genome is lacking sequence diversity. Such profound differences in genetic diversity can be consequential, having been associated with drug resistance and adaptability, with the potential to affect filarial eradication.

## Author summary

Almost a billion people receive >7.7 billion doses of treatment aimed at eliminating lymphatic filariasis, which is caused by three filarial nematodes: *Wuchereria bancrofti*, *Brugia malayi*, and *Brugia timori*. Drug resistance and adaptation are both associated with pathogen success as well as higher levels of genetic diversity. In an examination of genetic diversity in *Brugia malayi* and *Brugia pahangi*, we observed a lack of genetic diversity over a third of the genome that is found on chromosome X. These species have neo-X chromosomes where a chromosome X fused with an autosome. Using publicly-available published data, the other filarial nematodes of greatest human significance are also found to have a similar lack of genetic diversity on their neo-X chromosomes. The two filarial nematodes with publicly-available data that lack neo-X chromosomes did not have this lack of genetic diversity. This lack of sequence diversity in *B*. *malayi*, *W*. *bancrofti*, and *O*. *volvulus* could have profound effects on all traits encoded on chromosome X.

## Introduction

*Brugia malayi*, *Wuchereria bancrofti*, and *Brugia timori* are filarial nematodes (roundworms) that are responsible for lymphatic filariasis in humans with almost a billion people receiving >7.7 billion doses of treatment through lymphatic filariasis elimination efforts [1]. All filarial nematodes undergo a complex reproductive cycle that includes multiple larval stages within an arthropod vector followed by more larval stages, sexual development, and reproduction in vertebrate hosts [2]. Of the three filarial species responsible for human lymphatic filariasis, only a subset of *B*. *malayi* strains can be maintained in small animals in the laboratory, a prerequisite for rigorous laboratory-based studies. These laboratory populations are critical to our understanding of filarial biology, and are commonly used for anti-filarial drug trials [3]. *Brugia pahangi* can also be maintained in a laboratory life cycle, infects cats and dogs, and is occasionally zoonotic. *B*. *pahangi* and *B*. *malayi* use mosquito insect vectors and can co-infect dogs and cats [4]. Male *B*. *malayi* and female *B*. *pahangi* can produce viable offspring following mating in laboratory conditions [5,6], but the extent to which this happens successfully in nature is unknown. In addition to lymphatic filariasis, filarial nematodes are responsible for other diseases of medical and veterinary important, including human onchocerciasis [7] caused by the filarial nematode *Onchocerca volvulus*, human loiasis [8] caused by *Loa loa*, and dog and cat heartworm caused by *Dirofilaria immitis* [9].

*Onchocerca volvulus* [10], *Brugia malayi* [11–13], and *Brugia pahangi* [14] all have nearly complete genomes with chromosome-level assemblies of autosomes and chromosome X, while chromosome Y has yet to be resolved in any filarial nematode. Draft genomes are available for many other filarial nematodes [15], including *W*. *bancrofti* [16], *L*. *loa* [17], and *D*. *immitis* [18]. The genomes of all filarial nematodes are represented by six Nigon elements [12,19,20] that reflect conserved chromosomal segments that likely reflect the ancestral chromosome state in many nematodes, similar to Muller elements in *Drosophila* species [21]. In the case of filarial nematodes, the composition of these elements was primarily determined through homology to the completed genomes of *O*. *volvulus*, *Caenorhabditis elegans*, and/or *B*. *malayi* [12,19,20].

An important resource for filarial nematode research is the Filariasis Research Reagent Resource Center, better known as FR3, which maintains both *B*. *malayi* and *B*. *pahangi* worms across the life cycle in both Mongolian gerbils (jirds; *Meriones unguiculatus*) and cats [3]. At FR3, *B*. *malayi* and *B*. *pahangi* are passaged in cats via a mosquito vector. First, blood containing microfilariae is drawn from multiple cats, and pooled together. Then, this pooled blood is fed to mosquitos to allow microfilariae to develop to infective third-stage larvae (L3) which are extracted from mosquitos and introduced into an uninfected cat. Not all mosquitos survive infection with microfilariae, and not all infective L3 worms that are introduced into cats mature into viable adults. Infective L3s are also used to inoculate Mongolian gerbils that are used as a source of much of the material that is distributed by FR3. There are several steps where bottlenecks could occur, and different labs that maintain the life cycle have their own methods to prevent bottlenecks.

Genetic diversity can be influenced by bottlenecks, polyandry, population size, sex-biased population size, sex-biased or sex-exclusive inheritance, the rate of recombination, the mutation rate, and selection [22,23]. Bottlenecks occur when there is a rapid reduction in the population size such that allele frequencies shift dramatically [24] and have been studied in other parasite species [25–27]. These bottlenecks can significantly reduce genomic variation, but the presence of alleles that confer survival advantages can also generate selective sweeps that produce similar reductions in genomic variation [28]. Sex chromosomes add additional complexity to genetic diversity. For instance, in heteromorphic sex chromosomes like those in X-Y sex determination systems (which includes some filarial nematodes), the X chromosome has reduced genetic diversity by virtue of reduced effective population size. In a population with random mating (e.g. one without polyandry), this results in ~0.75 variance on chromosome X and ~0.25 variance on chromosome Y relative to the autosomes, but in species with multiple mating, this variance can be reduced even further [29].

Though multiple centers across the globe maintain *B*. *malayi* in laboratories, many of these laboratory populations are derived from the same initial population. Several cats were experimentally infected in the early 1960s with a sub-periodic zoophilic *B*. *malayi* strain that is reported to be derived from a human patient from Malaysia [30] and distributed to numerous places by Prof. Dr. C. P. Ramachandran [31,32]. Recipients included the Central Drug Research Institute, Lucknow, India, and the University of California Los Angeles (UCLA), among others. Most modern *B*. *malayi* laboratory lines are descended from this latter line at UCLA [3], including populations maintained and distributed by TRS labs and the NIAID-funded Filariasis Research Reagent Resource Center (FR3). FR3 and TRS supply one another worms when either laboratory has issues with their populations. In addition, investigators acquire worms from FR3 and/or TRS to establish their own culture collections and replenish with worms as needed, including the laboratories of Prof. Mark Taylor and Dr. Joseph Turner in the Liverpool School of Tropical Medicine and Dr. Gary Weil and Dr. Ramakrishna Rao at Washington University in St. Louis. A further *B*. *malayi* line was established independently from an infected woman in Narathiwat Province, southern Thailand, and has been maintained at The Faculty of Tropical Medicine, Mahidol University, Bangkok, then Chiang Mai University, Thailand, for ~40 years with no mixing with the other laboratory lineages [33].

The *B*. *pahangi* lineage at FR3 is thought to have been established in the 1970s [34] from a green leaf monkey. Because *B*. *pahangi* and *B*. *malayi* share very similar life cycles, the procedure for laboratory maintenance for both species at FR3 is similar.

Using samples of *B*. *malayi* and *B*. *pahangi* from multiple laboratory centers as well as natural samples of *B*. *pahangi* that were acquired from wild cats [35], we sought to investigate the genomic diversity within these *Brugia* populations. Given the potential for frequent bottlenecks both in nature and the laboratory, there is the repeated and significant risk of a founder effect that we sought to examine. To this end, we have employed public data from other filarial nematodes, including *W*. *bancrofti*, *L*. *loa*, *O*. *volvulus* and *D*. *immitis* in order to place this population diversity in the context of the broader filarial nematode family.

## Materials and methods

### *B*. *malayi* library preparation and sequencing

Adult male worms were provided from the following *B*. *malayi* centers: Washington University in St. Louis, MO, USA; Liverpool School of Tropical Medicine, UK; TRS Laboratories, Athens, GA, USA; FR3, Athens, GA, USA; Central Drug Research Institute, Lucknow, India; and Chiang Mai University, Chiang Mai, Thailand (**S1 Text**). Adult male worms were sequenced, since females are typically gravid precluding obtaining their individual genome. While virgin females would be a viable alternative, the difficulties in isolating them would have precluded us from obtaining many of the samples used here. Frozen single adult males recovered from the host gerbil were homogenized separately in 50 μl Buffer G2 from the genomic DNA buffer set (Qiagen) supplemented with RNase A (Qiagen) to 200 μg/mL. Homogenization was performed in a 1.5 mL microcentrifuge tube using a disposable micro pestle (Kimble-Chase). The homogenate was removed to a fresh tube and then the pestle and original tube were washed with an extra 0.95 mL of G2 buffer with RNase which was then added to the sample. The homogenized sample was then processed according to the protocol for tissue samples described in the genomic DNA handbook (Qiagen) and using genomic-tip 20/G gravity flow columns (Qiagen) except 80 U proteinase K (New England Biolabs) were used. Elution buffer QF was prewarmed to 50°C to increase DNA recovery. The DNA was precipitated by centrifugation as recommended, but in the presence of 20 μg glycogen (Invitrogen). Genomic DNA was sheared to ~380 bp with an ultrasonicator (Covaris) and used to construct indexed PE Illumina libraries using the NEBNext Ultra DNA kit (New England Biolabs). All samples were sequenced on the Illumina HiSeq 2500 with a read length of 100 bp, except for W_male_2 and W_male_6, which were sequenced on the Illumina HiSeq 4000 with a read length of 150 bp. While the data was generated specifically for this study, the data from a subset of samples were used in a previously published study to aid in identification of sex chromosomes and as such these methods are previously described for those samples [12].

### *B*. *pahangi* library preparation and sequencing

Adult *B*. *pahangi* male worms were provided from the following locations: FR3 laboratories, at both University of Georgia, Athens, GA, USA; University of Wisconsin, OshKosh, WI, USA (**S1 Text**) and University of Malaya, Kuala Lumpur, Malaysia [35]. Adult females were obtained from FR3 laboratories and pooled for the purposes of this analysis. Pooled adult female samples were prepared as described in Mattick et al [14]. Endemic isolates from Malaysia were prepared in an identical fashion to the *Brugia malayi* samples described above. Frozen single adult males obtained from FR3 and recovered from the same host gerbil were separately homogenized under liquid nitrogen in 1.5 mL microcentrifuge tubes. The samples were processed according to the Qiagen DNeasy blood and tissue insect protocol using 180 μl buffer ATL and 20 μL proteinase K. The samples were processed according to the manufacturer’s recommendations and eluted in 200 μL of buffer AE. After DNA isolation, the pooled adult female sample and the *B*. *pahangi* male FR3_UWO_Bp1AM_09 sample were sequenced on the Illumina HiSeq2500 from KAPA Hyper libraries with 150 bp paired-end reads. For all other *B*. *pahangi* samples, genomic DNA was sheared to ~380 bp with an ultrasonicator (Covaris) and prepared into an indexed, paired-end Illumina library using the NEBNext Ultra DNA kit. These samples were sequenced on the Illumina HiSeq 4000 with 150 bp paired end reads.

### Sample variant calling and processing for all individual nematode species

Each individual *B*. *pahangi*, *B*. *malayi*, *O*. *volvulus*, *D*. *immitis*, *C*. *elegans* and *Drosophila melanogaster* sample was mapped against its respective genome (GCA_000002995.5, GCA_012070555.1, GCA_000002985.3, GCA_001077395.1, GCA_000499405.2, GCA_000001215.4) [14,36–40] using BWA MEM [41] with the following settings: -M -a. The resulting BAM files were all sorted and de-duplicated using the Picard tools SortSam and MarkDuplicates, respectively [42] using default parameters for both. Single Nucleotide Variants (SNVs) were jointly called for each sample using Genomic Variant Call Format (GVCF) files generated using the Genome Analysis Tool kit (GATK) [43] with the HaplotypeCaller with the--read-filter MappingQualityReadFilter setting. The resulting GVCF files were merged and jointly called for SNVs using the GATK GenomicsDBImport and GenotypeGVCFs functions, then filtered using a manual filter with the following settings:--filter-name "QD"--filter-expression "QD < 5.0"--filter-name "QUAL"--filter-expression "QUAL < 30.0"--filter-name "DP"--filter-expression "DP < 14.0"--filter-name "MQ"--filter-expression "MQ < 30.0"--filter-name "MQRankSum"--filter-expression "MQRankSum < -12.5"--filter-name "ReadPosRankSum"--filter-expression "ReadPosRankSum < -8.0"--filter-name "FS"--filter-expression "FS > 60.0". For male samples from species where chromosome structure was known (*B*. *malayi*, *B*. *pahangi*), the autosomes were called with a ploidy of 2, while the X chromosome was called at a ploidy of 1. For female samples from species where chromosome structure was known (*O*. *volvulus*), the autosomes and X chromosome were called with a ploidy of 2. Filtration in samples called with a ploidy of 1 were filtered with--filter-name "DP"--filter-expression "DP < 7.0" to reflect the reduced sequencing depth on those sequences. Putative known pseudoautosomal regions from *B*. *malayi*, *B*. *pahangi*, and *O*. *volvulus* were excluded from variant analysis.

### Sample variant calling and processing for multi-individual samples

Each multi-individual *W*. *bancrofti* sample was mapped against its respective genome (GCA_000002995.5, GCA_012070555.1) [14,37] using BWA MEM [44] with the following settings: -M -a. The resulting BAM files were all sorted and de-duplicated using the Picard tools SortSam and MarkDuplicates respectively [42] using default parameters for both. SNVs were called using the Freebayes software, specifically the freebayes-parallel feature using default parameters.

### SNV density and Pi analysis

SNV density can allow for the identification of regions of the genome that are under- or over-represented in variants relative to the entire genomic sequence. SNV density across each of the chromosomes was calculated over 10-kbp sliding non-overlapping windows, considered as 20,000 possible variant sites with homozygous variants counting for 2 site changes and heterozygous variants counting as 1 site change. Pi was calculated using VCFtools over 10 kbp non-overlapping windows for all samples with a genomic coverage > 80% (**S1 Table**) for samples with a ploidy of 2. Because VCFtools requires diploid sites, the R package PopGenome [45] was used with default parameters to calculate Pi for *B*. *malayi*, *B*. *pahangi* and *O*. *volvulus* X chromosomes. Plots of SNV density and Pi were generated using the ggplots2 package in R [46], with the 10-kbp regions as the X-axis and Pi as the Y-axis. A density plot for Pi for each species was generated using the geom_density function of ggplots with default settings on the 10-kbp values of Pi across each chromosome. SNV density and Pi were assigned to Nigon elements, which were determined as previously described [12]. Briefly, contigs were mapped against *B*. *malayi*, *O*. *volvulus* and *C*. *elegans* using the NUCmer tool from the MUMmer package v.3.23 [47], and contigs were assigned to a specific Nigon element based on the largest match against each specific Nigon element. Principal component analysis was conducted on all autosomal variants in *Brugia malayi* and *Brugia pahangi* individuals using PLINK v.1.9 [48] with the--pca parameter. The resulting primary two principal components for each species were plotted using the geom_point function of ggplots with default settings in R.

### Phylogenetic relationships

Phylogenetic relationships for chromosome X and the autosomes were developed by first obtaining current genomes for *B*. *timori*, *W*. *bancrofti* and *O*. *volvulus* from WormBase [49]. Conserved nematode genes from these genomes, in addition to *B*. *malayi* and *B*. *pahangi*, were predicted using BUSCO v.4.06 package and its nematoda_odb10 database [50]. To ensure orthology, the genomes that were not in chromosome form (i.e. *B*. *timori* and *W*. *bancrofti*) were aligned against *B*. *malayi* using the NUCmer tool from the MUMmer package v.3.23 [47]. Contigs were binned to a chromosome based on maximum match length, and genes were assigned to chromosome X or the autosomes based on their contig matches. Genes present in all 5 species were aligned using TranslatorX [51] and filtered to include only those that were <15% dissimilar (>85% similarity) at the amino acid level and had at most a difference of 10% in gene length amongst all 5 orthologues. This left a total of 38 genes on chromosome X, and 228 genes on the autosomes. Trees were generated for these sequences using IQ-TREE with default parameters [52], and plotted using iTOL [53]. Mitochondrial sequences (NC_004298.1, CM022469.1, NC_016186.1, AP017686.1) for each species were obtained from GenBank, and aligned at the nucleotide level using MAFFT v.7.427 [54]. The mitochondrial tree was generated and plotted in an identical manner to the autosome and chromosome X trees.

### Ethics statement

All animals in the US were handled in accordance with guidelines defined by the Animal Welfare Act (A3381-01), Association for Assessment and Accreditation of Laboratory Care International (AAAALAC), PHS Policy for the Humane Care and Use of Laboratory Animals, and the Guide for the Care and Use of Laboratory Animals. Animal work for FR3 was approved under the University of Georgia Athens Institutional Animal Care and Use protocol A2010 12–005 and A2013 11–009 or the University of Wisconsin OshKosh under IACUC protocol number 0026-000246-R2-01-12-17. All animal research at TRS was approved under Institutional Animal Care and Use Protocol 13–03 or 14–03. All animal work at WUSM was approved under WUSM Institutional Animal Care and Use Protocol 20120025.

The study in Lucknow India bears IAEC approval number 129/08/Para/IAEC/renew (84/09) dated April 27, 2009.

All experiments on animals at Liverpool School of Tropical Medicine were approved by the ethical committees of Liverpool School of Tropical Medicine and the University of Liverpool and were conducted according to Home Office Legislation, the revised Animals (Scientific Procedures) Act of 1986 (project license numbers 3002974, P86866FD9).

Approval for using gerbils for sample work in Malaysia was granted by the University of Malaya Animal Care and Use Committee (Ref. No. PAR/29/06/2012/RM [R]).

The protocol for samples obtained from Thailand was approved by the Institutional Animal Care and Use Committee (Protocol Number 15/2562) of the Faculty of Medicine, Chiang Mai University, Chiang Mai province, Thailand.

## Results

### Genomic variation in *B*. *malayi* laboratory populations

Between 4–6 individual adult male *B*. *malayi* worms were sequenced from each of 6 laboratory populations, which are from three primary *B*. *malayi* population groups: (a) FR3 and FR3 derived lines, including the continually maintained FR3 line, the line maintained by TRS labs, and the lines at Washington University in St. Louis and the Liverpool School of Tropical Medicine; (b) those from a life cycle established at the same time as the FR3-derived lines, but maintained independently for decades in Lucknow, India; and (c) those from the life cycle in Chiang Mai, Thailand, established from a completely independent human infection and maintained in the laboratory independently for ~40 years. Paired-end Illumina sequencing reads were generated to an average of 85× sequencing depth from individual adult male *B*. *malayi* worms (**S1 Table**). These adult male worms from each site were collected from the same gerbil, with the exception of TRS, where half of the worms were obtained from a different host gerbil (**S2 Table**). All of the reads were mapped to the reference *B*. *malayi* genome [11–13] that was obtained with worms from FR3 and TRS. The *B*. *malayi* samples had an average of 105,264 SNVs per sample, and 21,227 insertions/deletions per sample identified with the GATK HaplotypeCaller called jointly on all samples. The *B*. *malayi* samples had a transition/transversion ratio (ts/tv) ranging from 2.10–2.60 (**S2 Table**).

### SNV density and Pi across the *B*. *malayi* genome

The analysis of SNV distribution using Pi was calculated over the Nigon elements associated with each chromosome. Nigon elements are regions of nematode genomes that likely reflect the ancestral five autosomes and a single sex chromosome. Nigon elements persist despite genome rearrangements because of the infrequency of recombination between chromosomes in nematodes [12,19,20]. These are similar to Muller elements in *Drosophila* [21] with Nigon elements being denoted as Nigon-A, Nigon-B, Nigon-C, Nigon-D, Nigon-E, and Nigon-X. The gene content on Nigon elements remains largely conserved even following neo-X chromosome evolution, like the fusion of Nigon-D and Nigon-X in *Brugia* spp. and Nigon-D and Nigon-E in *O*. *volvulus* [12].

**Fig 1:**
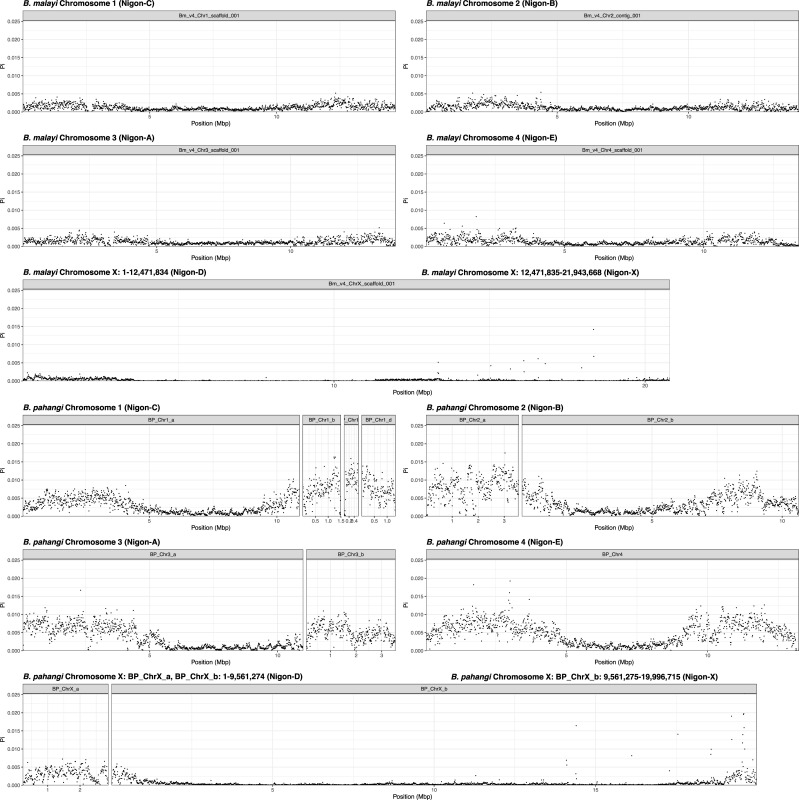
Pi across *B*. *malayi* and *B*. *pahangi* samples from multiple laboratory backgrounds. Pi was calculated across each of the *B*. *malayi* and *B*. *pahangi* contigs/scaffolds using VCFTools on a combined VCF file containing all samples. The results are organized by chromosome and Nigon elements. Chromosome X shows a distinct lack of nucleotide diversity relative to the autosomes. The lack of diversity on chromosome X appears to be present in nematodes from all laboratory centers for *B*. *malayi* and in both endemic and laboratory populations for *B*. *pahangi*. The plots for chromosome X are larger reflecting the increased size of chromosome X which is approximately twice the size of the autosomes. Chromosome Y is not resolved in either organism, and as such Pi could not be calculated.

**Fig 2:**
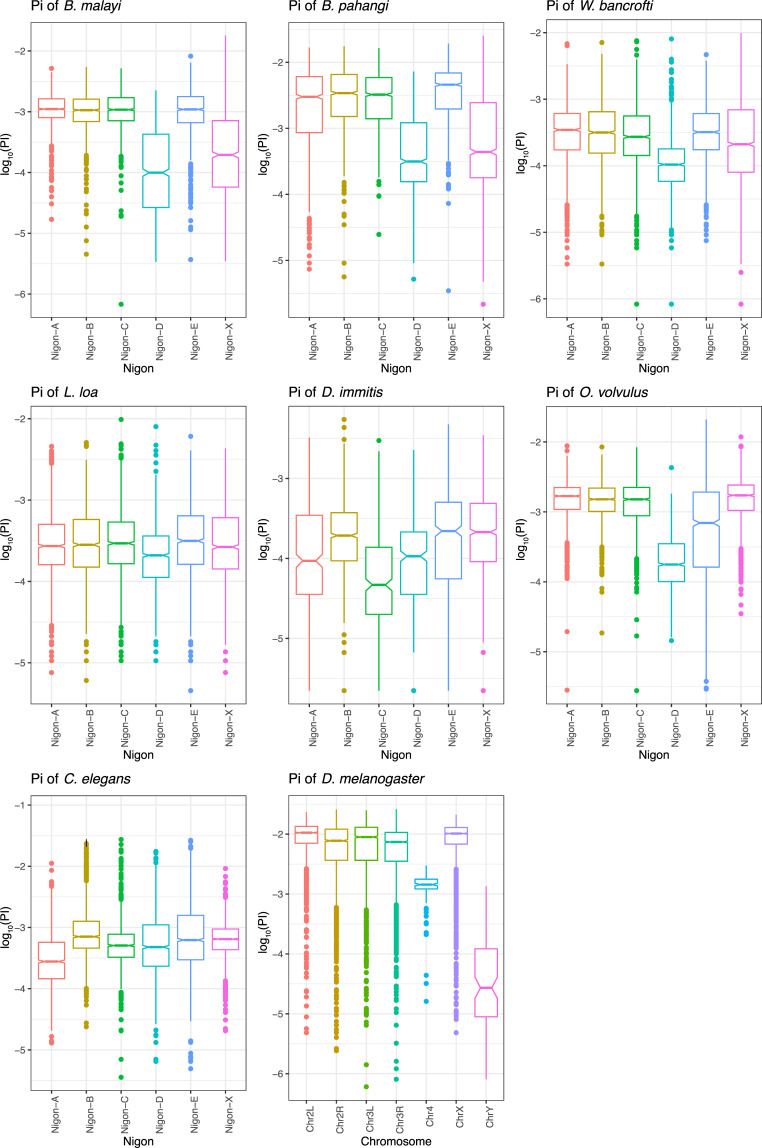
Pi across filarial nematode species and model organisms. Pi was calculated across *B*. *malayi*, *B*. *pahangi*, *W*. *bancrofti*, *O*. *volvulus*, *L*. *loa*, *D*. *immitis*, *D*. *melanogaster* and *C*. *elegans* using VCFTools on a combined VCF file containing all samples for each of those species. For all nematode species, contigs were assigned to a Nigon element based on their homology to *B*. *malayi*, *O*. *volvulus* and *C*. *elegans*. Values of Pi were log_10_-transformed to more readily visualize the distributions. Filarial nematodes with neo-X chromosomes (Nigon-D/Nigon-X in *Brugia* spp. and *W*. *bancrofti* and NigonD/Nigon-E in *O*. *volvulus*) have a significantly depressed Pi compared to autosomal Nigon elements or X chromosomes in other species (Nigon-D in *L*. *loa* and *D*. *immitis*, Nigon-X in *C*. *elegans*, and chromosome X in *D*. *melanogaster*). This suggests that the loss of diversity observed in *B*. *malayi* and *B*. *pahangi* are not limited to those species and related to the formation of the neo-X chromosome. Chromosome 4 in *D*. *melanogaster* also has a decrease in Pi; it is a small chromosome sometimes referred to as the dot chromosome that is largely heterochromatic and may formerly have been a sex chromosome [74].

The average SNV density across all samples **(S1 Fig)** and the amount of allelic diversity (Pi) for all 26 *B*. *malayi* samples **(Fig 1)** were similar when calculated in 10-kbp windows across each of the Nigon elements for each sample. For species where chromosome X and the pseudo-autosomal region were defined and the samples were known to be male (*B*. *malayi* and *B*. *pahangi*), Pi for this chromosome was calculated using a ploidy value of 1, while the remaining chromosomes were calculated using the standard ploidy of 2. In these cases, X-specific will refer to the region of chromosome X that is not shared with chromosome Y, while the pseudo-autosomal region will refer to the shared sequence between the X and Y chromosomes. After excluding the pseudo-autosomal region of chromosome X, the average Pi across the X specific Nigon-D and Nigon-X are 5-fold lower (π_X_/π_A_ = 0.19) when compared to similar regions of the autosomes **(Fig 2**).

**Fig 3:**
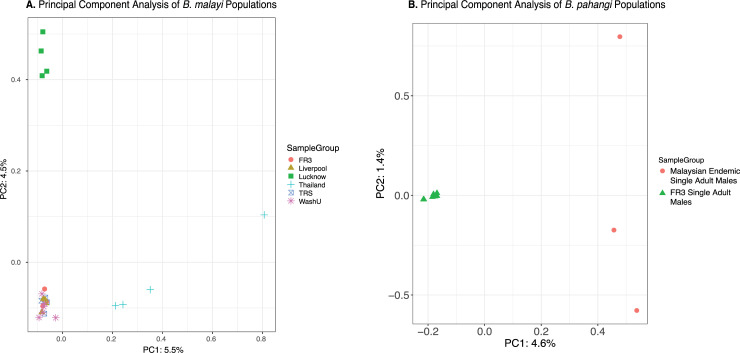
Principal component analysis of *B*. *malayi* and *B*. *pahangi* samples. Principal component analyses of the *B*. *malayi* **(A)** and *B*. *pahangi* **(B)** samples were conducted using PLINK with default parameters on each individual sample for each population, and the resulting outputs were imported into R and plotted using geom_point from ggplots. All of the FR3-derived *B*. *malayi* samples cluster very tightly together, except for those derived from the Lucknow strain, which are separated by principal component 2. Principal component 1 primarily divides the 4 samples from Thailand, which not only are distinct from FR3-derived worms, but are much more distinct from each other than FR3-derived worms are from each other. The FR3 single adult male *B*. *pahangi* all cluster together, while samples from wild infected cats from Malaysia appear to dominate the variation along both principal components.

A principal component analysis identified that while populations recently supplemented from the FR3 lineage are very similar, the Thai samples and the Indian samples are significantly different, despite those from Lucknow, India, sharing a common background with the FR3 lines (**Fig 3A**).

### Genomic variation in *B*. *pahangi* samples

Individual adult male *B*. *pahangi* worms were sequenced from endemic *B*. *pahangi* from a cat in Malaysia and from the *B*. *pahangi* FR3 laboratory population. For sequencing of endemic *B*. *pahangi*, *Aedes togoi* mosquitos were allowed to feed on a naturally-infected microfilaremic wild cat, L3s were recovered, and these L3s were used to infect gerbils as previously described by Lau et al. [35]; three of these adult worms from a single gerbil were individually sequenced and used for variant analysis. These three worms were compared to seven adult male *B*. *pahangi* worms from the FR3 laboratory population from two gerbils. All of these samples were sequenced on the Illumina HiSeq platform, resulting in an average 105× sequencing depth (range: 22×-217×) per individual across the genome (**S3 Table**). All samples were mapped to the *B*. *pahangi* FR3 genome [14]. On average there were 315,514 SNVs and 107,463 insertions/deletions identified with the GATK HaplotypeCaller in each *B*. *pahangi* sample with a consistent ts/tv of 2.67–2.95, which is higher than the ts/tv for *B*. *malayi* calculated above.

### SNV density and Pi across the *B*. *pahangi* genome

The average SNV density across all samples **(S2 Fig)** and the amount of allelic diversity (Pi) **(Fig 1)** for all 10 samples were calculated in 10-kbp windows across each of the Nigon elements for each sample. Based on both the sequencing depth (**S3 Fig**) difference between BP_ChrX_c and other contigs in the *B*. *pahangi* chromosome X and the decrease in apparent sequence diversity on chromosome X contigs in all but BP_ChrX_c (**S4 Fig**), BP_ChrX_c was determined to be the pseudo-autosomal region and analyses were adjusted accordingly. After excluding the pseudoautosomal region of the X chromosome, the average Pi across Nigon elements D and X is 5-fold lower (π_X_/π_A_ = 0.21) when compared to Nigon elements in the autosomes **(Figs 1 and 2**).

A principal component analysis using PLINK identified that the FR3 *B*. *pahangi* samples are distinct from the endemic samples, but that the FR3 samples are also much more closely related to each other than the endemic samples are to one another (**Fig 3B**). The second principal component primarily separates out each endemic sample, suggesting that these worms have significantly more diversity than those from the FR3 lineage.

### Introgression

**Fig 4:**
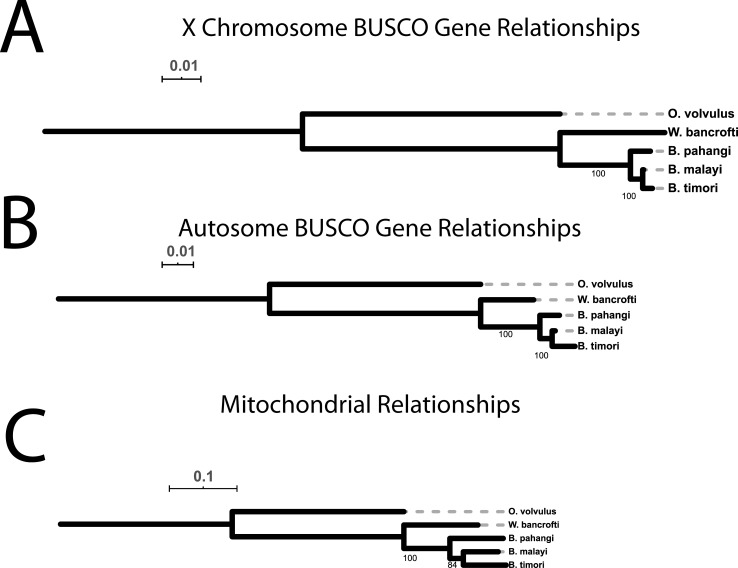
Phylogenetic trees of conserved nematode BUSCO genes and mitochondria between filarial species. Conserved genes predicted by BUSCO in *B*. *malayi*, *B*. *pahangi*, *W*. *bancrofti*, *B*. *timori* and *O*. *volvulus* were separated out by their location and divided based on their presence on chromosome X of *B*. *malayi* and *B*. *pahangi* **(A)** or the autosomes of those species **(B)**. These gene sets were used to construct phylogenetic trees using IQ-TREE (bootstrap = 1000) that were midpoint rooted in IQ-TREE (https://itol.embl.de/). **(C)** Mitochondrial genome sequences of these organisms were aligned via MAFFT, and trees were generated via IQ-TREE. The relationships between filarial species consistently show *B*. *malayi* and *B*. *timori* as more closely related to each other than to *B*. *pahangi* such that any loss of chromosome X diversity likely predates the divergence of the three organisms.

In each *Brugia* nematode, there are three genomes—the mitochondrial genome, the *Wolbachia* endosymbiont genome, and the nuclear genome. Because of the similarities in nucleotide identity, chromosome structure (including a largely shared X chromosome and similar pseudoautosomal region) and genome size between *B*. *pahangi* and *B*. *malayi*, as well as the documented ability for these species to successfully cross [5], we tested if there was introgression between *B*. *pahangi* and *B*. *malayi*. If an introgression occurred that resulted in the transfer of a chromosome X from one *Brugia* species to the other, one would expect that a phylogenetic tree drawn from chromosome X would look different than that of the autosomes. However, phylogenetic trees of a subset of conserved genes on the autosomes of these agents of lymphatic filariasis and a related filarial parasite, *Onchocerca volvulus*, are similar in topology and relative distance when compared to those on chromosome X and the mitochondria, while the rates of variation are different (**Fig 4**). These phylogenetic patterns between *B*. *malayi* and *B*. *pahangi* that are the same for chromosome X, the autosomes, and mitochondrial sequences suggest that the decreased variation on chromosome X did not result from introgression. The conserved phylogenetic topology suggests that this lack of sequence diversity predates the origins of *Brugia* spp.

### Other filarial genomes

To examine the loss of sequence diversity on chromosome X more widely, particularly with respect to the two neo-X chromosomes, we compared the sequence diversity across exemplar filarial nematodes that have sequence data from multiple samples, including *B*. *malayi*, *B*. *pahangi*, *O*. *volvulus* [10], *W*. *bancrofti* [16], *L*. *loa* [17], and *D*. *immitis* [18]. These analyses capitalized on the organization of nematode genomes that allows for the attribution of contigs to Nigon elements even in the highly fragmented genomes like *W*. *bancrofti* [16], *L*. *loa* [55] and *D*. *immitis* [56]. *W*. *bancrofti* is predicted to have a Nigon-D and Nigon-X fused neo-X chromosome like *Brugia* spp., *O*. *volvulus* has a Nigon-D and Nigon-E fused neo-X chromosome, and *D*. *immitis* and *L*. *loa* are predicted to have just Nigon-D as their chromosome X [12]. If the loss of sequence diversity in chromosome X of *Brugia* is associated with neo-X chromosome evolution, we would expect there to be a similar loss in the phylogenetically distinct *O*. *volvulus* that we do not see in *D*. *immitis* or *L*. *loa*. In addition, the results were compared to similar data [57,58] for the model organisms *C*. *elegans* and *D*. *melanogaster* that have complete genomes [59,60], and a large amount of available population data. *C*. *elegans* is a free-living nematode with an XO reproductive system, while *D*. *melanogaster* is an arthropod with an XY reproductive system.

Publicly-available WGS data from populations of *O*. *volvulus* (mixed sex individuals), *W*. *bancrofti* (mixed samples), *L*. *loa* (mixed samples), *D*. *immitis* (individual males), *C*. *elegans* (mixed sex individuals), and *D*. *melanogaster* (mixed sex individuals) were analyzed to ascertain whether the loss of diversity observed in *B*. *malayi* and *B*. *pahangi* was present in other filarial nematodes. Given the fragmented nature of some of the filarial nematode genomes and the lack of Y chromosomes in some species, the pseudo-autosomal region could only be excluded from *O*. *volvulus* and *D*. *melanogaster*. Contigs from the nematode genomes were assigned to Nigon elements based on their homology to *B*. *malayi*, *C*. *elegans*, and *O*. *volvulus*. The distribution of Pi across Nigon elements was non-normal with a mean outside the interquartile range such that the data violates many of the assumptions of common statistical tests. However, visual inspection of the box plots reveals that in nematodes with neo-X chromosomes (i.e. *Brugia* spp., *W*. *bancrofti*, and *O*. *volvulus*) chromosome X can clearly be delineated with a lower Pi (**Fig 2**), despite the difference in the Nigon-composition of those neo-X chromosomes. In contrast, in nematodes without neo-X chromosomes (i.e. *D*. *immitis*, *L*. *loa*, and *C*. *elegans*) as well as in *D*. *melanogaster*, chromosome X cannot be clearly delineated (**Fig 2**), and Pi on chromosome X is in line with Pi on the autosomes. This indicates that this profound lack of sequence diversity on chromosome X is not due solely to the life cycle and lifestyle of filarial nematodes, but instead to creation of neo-X chromosomes through fusion with an autosome.

## Discussion

*B*. *malayi* and *B*. *pahangi* filarial nematodes populations have genetic diversity that is consistent with the known separation over time of these populations (**Fig 3**). The greatest difference is seen between endemic nematodes and laboratory populations in the case of *B*. *pahangi*, or between independently derived laboratory populations in the case of *B*. *malayi*. To a lesser extent there are differences between nematodes that were derived from the same human sample but have been maintained separately for decades reflected in the differences between Lucknow and the FR3 samples.

Lack of access to clinical samples precluded their inclusion in this study. While the passage of laboratory populations through non-native hosts could impact the genetic diversity, introducing new bottlenecks and selective pressures, the lack of diversity on neo-X chromosomes was found in at least two populations for each of four species with known neo-X fusions (*B*. *malayi*, *B*. *pahangi*, *W*. *bancrofti*, and *O*. *volvulus*) and was absent from the two filarial nematodes that lack such fusions (*L*. *loa* and *D*. *immitis*). Further population level data and the completion of filarial nematode genomes will likely shed further light on the factors influencing genetic diversity in filarial nematodes as well as parasitic nematodes more broadly.

A significant difference in genetic diversity was observed between autosomes and chromosome X. Genetic diversity can be influenced by bottlenecks, polyandry, rate of recombination, mutation rate, selection, and effective population size [22,23]. The loss of genetic diversity on chromosome X is not limited to just laboratory populations (and the bottlenecks associated with laboratory propagation) since natural populations of *W*. *bancrofti* and *B*. *pahangi* have the same loss of diversity. Although polyandry and population shrinkage may also contribute to loss of diversity in filarial nematodes, it is quite likely to be similar for all of the examined filarial nematodes given their life history.

The rate of recombination is expected to be suppressed in sex chromosomes relative to autosomes [61], which is supported by the significant reduction in intrachromosomal inversions observed in the *Brugia* chromosome X relative to its autosomes [12]. In addition, chromosome Y has an abundance of repeats and transposable elements that prevented its assembly [12], and these repetitive elements are predicted to play a critical role in the further suppression of recombination [62].

In mammals and birds, the higher mutation rate in males over females leads to differences in the mutation rate between autosomes and sex chromosomes [63], while in at least one plant [64] the autosome and sex chromosome mutations are approximately equal. Differences in mutation rate on the sex chromosomes in mammals are associated with more rounds of replication in male gametes, which is likely also the case in filarial nematodes. However, we expect male gametogenesis to be similar between all examined filarial nematodes, such that the differences we observe are not likely attributed to the mutation rate.

Genetic diversity can also be influenced by sex-biased effective population size, sex-biased inheritance, and sex-exclusive inheritance [22,23]. While we cannot rule out the effects of sex-biased inheritance or sex-exclusive inheritance, we suggest that they would likely be the same across all examined filarial nematodes.

Across nematodes and even filarial nematodes, there is a diversity of sex chromosomes, with XO sex determination being common, but XY being present, and even some nematodes having three sexes [65]. Among the filarial nematodes examined, *L*. *loa* and *D*. *immitis* are thought to be XO [66], with *Brugia* spp. and *Onchocerca* spp. being XY [66] resulting from different neo-X fusions [12]. In the absence of selection and no sex bias in reproduction, the expected population size for an organism with heteromorphic XY chromosomes, like *Brugia* and *Onchocerca* filarial nematodes, the autosome:(chromosome X):(chromosome Y) allelic frequency is 4:3:1. As a consequence, a reduction of nucleotide diversity is expected on heteromorphic sex chromosomes, with π_X_/π_A_ ~ 0.75 [10,22]. Similarly, nematodes with XO sex determination would have an expected autosome:(chromosome X):(chromosome Y) allelic frequency of 4:3:0 with π_X_/π_A_ ~ 0.75. However, we observe π_X_/π_A_ ~ 0.2 for both *Brugia* species.

**Fig 5:**
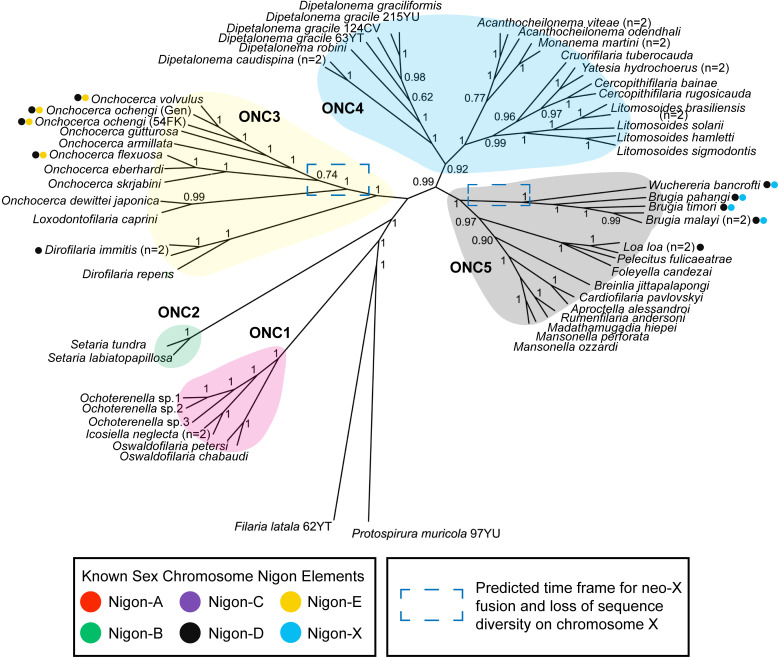
Phylogenetic relationships related to sex chromosome Nigon content. The phylogenetic relationship of filarial nematodes is shown as adapted from Lefoulon et al. [75]. Nigon element assignments for the sex chromosomes are shown when known or inferred previously [12]. The loss of diversity on the sex chromosome co-occurs with the instances of chromosomal fusions between Nigon-IV and Nigon-X in ONC5 and between Nigon-IV and Nigon-V in ONC3, but does not appear to be present in species that do not contain the chromosomal fusion in either the ONC3 or ONC5 clades.

Upon examination of other filarial nematodes, a reduction in π_X_/π_A_ similar to that in *Brugia* spp. was observed for *W*. *bancrofti* and *O*. *volvulus*, all four of which have neo-X chromosomes that emerged after fusion of chromosome X with an autosome. In the case of filarial worms, different neo-X chromosomes were formed at least twice by the fusion of two Nigon elements [12,19,20]. The common Nigon element in these fusion events appears to be Nigon-D, which is likely the ancestral sex chromosome of filarial nematodes [12,19,20]. The chromosomal fusion event in the ONC3 clade, containing *Onchocerca* spp., joined Nigon-D and Nigon-E, while the chromosomal fusion in the ONC5 clade, containing *Brugia* spp. and *Wuchereria* sp., joined Nigon-D and Nigon-X (**Fig 5**). Both times that there is a loss in diversity on chromosome X in this study, there is a concomitant neo-X fusion. And conversely, where there is not a neo-X fusion, there is not the loss of diversity (i.e. *L*. *loa* and *D*. *immitis*). As such this lack of genetic diversity on chromosome X seems consistent with the formation of the neo-X chromosomes prior to several speciation events, like that of *Brugia* spp. and *W*. *bancrofti* (**Fig 5**). Chromosomal fusion events are known to reduce genomic diversity in species as the effective population size of the sex chromosome is reduced and novel genes and dosage mechanisms must be generated to compensate for the fusion [67,68]. For example, in *Sylvoidea* bird species, a loss of diversity on chromosome Z (the equivalent of chromosome X in ZW systems) is attributed to a neo-sex chromosome fusion [69].

Chromosomal fusions may not be the only source of diversity loss on chromosome X. For example, *Haemonchus contortus*, a parasitic nematode, does not show evidence of a recent chromosomal fusion. Yet the *H*. *contortus* π_X_/π_A_ is 0.36 [70], which is also lower than neutral expectation of π_X_/π_A_ ≃ 0.75. This decrease in *H*. *contortus* was attributed to host sex biases due to reproductive fitness being over-dispersed between males and females from polyandry and high fecundity [70]. However, filarial nematodes only seem to have this lack of genetic diversity on neo-X chromosomes despite likely polyandry and high fecundity across many or most filarial nematodes.

In nematodes, there has also been a transition in the sex chromosomes. Nigon-D is likely the ancestral chromosome for all Rhabditida nematodes, with a conversion of Nigon-X to chromosome X in Rhabditina nematodes, which includes *C*. *elegans* [12]. This transition does not appear to be associated with a difference in genetic diversity for chromosome X upon comparisons of *C*. *elegans* and the filarial nematodes without neo-X fusions, like *D*. *immitis* and *L*. *loa*. (**Fig 2**). It is possible that altering the sex determining Nigon element is not enough to cause diversity loss, and that it is specifically associated with chromosomal fusion. Alternatively, it is possible that enough time has elapsed to eliminate the signature associated with that transition at least with the resolution with which it was examined here.

The same processes that subject chromosome X to decreased genetic diversity and Muller’s ratchet also affect chromosome Y to a much larger degree [63,71]. In filarial nematodes, we do not have an assembled chromosome Y, and are limited to male-specific contigs attributed to chromosome Y. But the high repetitiveness of the sequences [12] suggests that filarial nematode Y chromosomes are undergoing a degeneration consistent with neo-Y formation.

Although chromosomal fusions appear to be associated with diversity loss in filarial worms, it is not yet clear if this will be found universally in other parasitic nematodes. This lack of chromosome X genetic diversity is important since most medically important filarial nematodes have neo-X fusions with a third of all genetic material being on chromosome X, representing a substantial loss of sequence diversity. Genetic material on chromosome X also undergoes recombination at a lower rate than the rest of the genome [61]. Thus the sex chromosome is more susceptible to Muller’s Ratchet [72], which is a process whereby deleterious mutations accumulate in the absence of recombination. This loss of diversity on such a large portion of the genome could have significant consequences. In other parasites, drug resistance and adaptability are associated with a higher level of genetic diversity, and its absence can prevent an organism from developing strategies of coping with adverse events [73].

## Conclusions

Populations were examined that were derived from two independent isolates of *B*. *malayi* and *B*. *pahangi*. For *B*. *malayi* this includes several populations derived from a human from Malaysia and a population from an infected woman in Thailand. For *B*. *pahangi* this includes the populations derived from a green leaf monkey from Malaysia and from naturally infected Malaysian cats. We observe a profound lack of sequence diversity on chromosome X in all independent populations of *B*. *malayi* and *B*. *pahangi* that is consistent with reduced chromosome X diversity in other sequenced filarial nematodes with neo-X chromosomes. Given the importance that sequence diversity has with respect to adaptability and the size of chromosome X, which is a third of the genome, this lack of sequence diversity in a third of the genome in medically important filarial nematodes is likely to have a large effect on the evolutionary trajectory of these species.

## Supporting information

S1 Fig *B*. *malayi* variant distribution across samples from multiple laboratory backgrounds. SNV density was calculated across each of the *B*. *malayi* chromosomes and averaged across all samples using 10 kbp windows across each contig and normalized to the total sample number (n = 26). Heterozygous variants were considered as half of the value of homozygous variants for the purposes of density calculations. There is a significant loss of variants in chromosome X that is consistent across all individual samples and is displayed here in aggregate, and the pseudoautosomal region of chromosome X is indicated by a red bar.
(EPS)

S2 Fig *B*. *pahangi* variant density. SNV density was plotted across each of the *B*. *pahangi* chromosomes averaged across all samples. Density was calculated using 10 kbp windows across each contig using R and normalized to the total sample number (n = 10). Heterozygous variants were considered as half of the value of homozygous variants for the purposes of density calculations. Similar to *B*. *malayi*, chromosome X of all of the *B*. *pahangi* samples show a significant lack of variation in the central region that is in contrast to the autosomes and the rest of chromosome X.
(EPS)

S3 Fig *B*. *pahangi* sequencing depth across all samples. Depth plots were calculated over 10 kbp non overlapping regions across the *B*. *pahangi* chromosomes. The predicted pseudoautosomal region (**S4 Fig**) has depth that is consistent with autosomal depth while the rest of chromosome X appears to be at half depth. This is consistent with a pseudo-autosomal profile in this contig.
(EPS)

S4 Fig Heterozygous *B*. *pahangi* SNV density across chromosome X. Density plots were generated for heterozygous SNV density values calculated over 10 kbp non-overlapping regions across the *B*. *pahangi* chromosome X. Pseudo-autosomal regions in chromosome X of *B*. *malayi* have been previously described [12], and an analysis of heterozygous SNVs in chromosome X of adult *B*. *pahangi* males (which should only be possible in pseudoautosomal regions) reveals that a similar region of the chromosome has an enriched value of Pi, indicating that *B*. *pahangi* has a similar pseudoautosomal region to *B*. *malayi*.
(EPS)

S1 Table Coverage and Sequencing Depth Metrics for all Samples in Fig 2. (XLSX)

S2 Table Metadata, Mapping statistics, and Variant Calls for *Brugia malayi*. (XLSX)

S3 Table Metadata, Mapping statistics, and Variant Calls for *Brugia pahangi*. (XLSX)

S1 Text Standard operating procedure for *Brugia* rearing in jirds across all Centers. This document includes all standard operating procedures for maintaining the laboratory life cycle for *B*. *malayi* and/or *B*. *pahangi* for the different laboratories that provided samples from their collections.
(DOCX)
