# Supplementary material for: X-treme loss of sequence diversity linked to neo-X chromosomes in filarial nematodes
Source: PLoS Negl Trop Dis. 2021 Oct 27;15(10):e0009838. doi: 10.1371/journal.pntd.0009838 (PMC8575316; doi:10.1371/journal.pntd.0009838)
Supplement: S1 Text — This document includes all standard operating procedures for maintaining the laboratory life cycle for B. malayi and/or B. pahangi for the different laboratories that provided samples from their collections. (DOCX) [file pntd.0009838.s008.docx]

**Standard Operating Procedures for Obtaining *B. malayi* Specimens for this Study from FR3**

All animals at NIAID FR3 were handled in accordance with guidelines defined by the Animal Welfare Act (A3381-01), Association for Assessment and Accreditation of Laboratory Care International (AAAALAC), PHS Policy for the Humane Care and Use of Laboratory Animals, and the Guide for the Care and Use of Laboratory Animals. Animal work was approved under the University of Georgia Athens Institutional Animal Care and Use Protocol A2010 12-005 and A2013 11-009.

All SOPs used by FR3 for rearing *B. malayi* and *B. pahangi* are available at http://www.filariasiscenter.org/protocols/Protocols.

**Standard Operating Procedures for Obtaining *B. malayi* Specimens for this Study from TRS Labs**

All animal research at TRS was approved under Institutional Animal Care and Use Protocol 13-03 or 14-03.

*B. malayi* nematodes were purchased from TRS Labs using Experimental infection protocol SOP 8.05 rv3, collecting infective larvae protocol SOP 8.04 rv6, and infecting mosquitoes SOP 8.03 rv5.

**Infecting *Aedes aegypti***

1. Allow approximately 600 female pupae to emerge as adults into a one-gallon carton as described in SOP 8.02.
2. Three to five-day-old mosquitoes are used for producing infective larvae.
3. Sugar cubes are removed from the cartons before the infective blood meal.
4. Blood is collected from an infected animal and heparinized, and a microfilarial count is made. If the blood contains too many microfilariae, it is diluted with normal heparinized blood. The best results are obtained using the following concentrations of microfilariae in the membrane feeder:
   - - 1. *B. pahangi* 100-200 mf/20µl
       2. *B. malayi* 80-100 mf/20µl
       3. *D. immitis* 30-50 mf/20µl
     1. If the counts are lower than these values, low numbers of infective larvae will be produced.
5. Mosquitoes are allowed to feed on infected blood maintained at approximately 37°C in a membrane feeding apparatus. This usually requires 1-2 hours. With *D. immitis*, it is important that fresh blood (collected within one hour of infection) be used for infecting each carton of mosquitoes.
6. Mosquitoes may also be infected by feeding on an infected animal (for details, see other SOPs).
7. After infection, sugar cubes and cotton containing water are placed on the nylon screen. Water is added to the cotton as needed, and the cotton is replaced every 5-7 days.
8. The cartons are covered securely with another piece of nylon screen as a secondary precautionary measure.
9. Infective larvae of *B. pahangi, B. malayi,* and *D. immitis* are collected no earlier than 11, 14, and 14 days, respectively, after the infective blood meal.

**Collecting Infective Larvae from *B. malayi***

1. Remove sugar cubes and water-soaked cotton from the cartons used to raise mosquitoes as described in SOP 8.2.
2. Remove dead mosquitoes by aspiration from the carton.
3. Place cotton containing ether on the nylon screen and then place the carton in a plastic bag for several minutes. This procedure must be done under a explosion-proof hood. Alternatively, the mosquito cartons may be placed in a freezer for approximately one minute.
4. After all mosquitoes are immobilized, remove the top of the carton and transfer the infected mosquitoes (maximum of 2,250 mosquitoes) to a piece of glossy paper (or similar smooth material). Replace the screened lid of the carton in case infected mosquitoes remain in the carton.
5. Quickly put the infected mosquitoes into a mortar without solution and gently crush them. Add 2 to 3 ml of chilled (5 to 10°C) Hanks' balanced salt solution (HBSS, pH 7.0) containing pen-strep (final concentration= 0.4 units penicillin/ml, 0.4 mcg streptomycin/ml, PS) to the mortar and gently crush the mosquitoes again. Unless collecting *D. immitis* for in-vitro assay, the addition of pen-strep to the HBSS is optional.
6. The crushed mosquitoes on the pestle and in the mortar are then rinsed with the chilled HBSS-PS or HBSS onto a 100 mesh sieve contained in a plastic petri dish.
7. The sieve containing the crushed mosquitoes is gently, but quickly, agitated to removed scales, eggs, and debris body parts (e.g., legs) of the mosquitoes. This procedure is repeated 3 to 4 times, using several dishes of fresh, chilled HBSS-PS or HBSS. As approximately 10% of the larvae may remain in these washings, it is advisable to keep these dishes until a sufficient number of larvae have been collected, i.e., it may be necessary to use all of the larvae from the washings to obtain a sufficient number of larvae.
8. The sieve is then removed to fresh, warm (initially 30-37°C) HBSS-PS or HBSS to allow the larvae to migrate out of the mosquitoes; the sieve is transferred to petri dishes containing fresh HBSS-PS or HBSS every 15 to 30 minutes. If collecting *D. immitis* for bioassay, the sieve is soaked in warm RPMI-1640 containing 300 units/mL Penicillin, 300mcg Streptomycin, 200 µg/mL Gentamicin, and 2.5 µg/mL Amphotericin B.

(Note: most of the *Brugia* larvae will have migrated out of the mosquitoes within about 2 hours. Larvae of *D. immitis* are less active, therefore, it is necessary to allow 1 to 2 more hours for collecting these larvae.)

1. When inoculating *Brugia* larvae intraperitoneally into jirds, the solution must be quite clean, i.e., free of debris and not "cloudy," to avoid peritonitis and possibly death of the jirds. Thus, the solution should be relatively "clean" before inoculation of larvae either SC or IP, but particularly before IP injections.

**Experimental Infection of Dogs, Cats, Ferrets, and Rodents with *B. malayi***

1. A stereo dissecting scope and a micropipette (i.e., modified pasteur pipette) are used to remove the infective larvae (L_3_) from the petri dishes.
2. The L_3_ are counted into separate petri dishes for each individual animal. The number of L_3_/animal will vary according to species of parasite and animal and the purpose of the study.
3. After counting is completed, the petri dish containing the L_3_ is tilted slightly to allow the L_3_ to settle to the lowest part of the dish.
4. Excess fluid is removed from the dish using a pasteur pipette, such that less than 1 ml of fluid (containing L_3_) remains in the dish.
5. The fluid containing the L_3_ is pulled up into a 1-cc tuberculin syringe.
6. For infecting rodents intraperitoneally, inject larvae into the peritoneal cavity as described in 2.2.1. A 1.0- or 1.5-inch needle may be used.
7. Inoculum size and route of infection may vary by study protocol objective and requirements.
8. Guidelines for establishing infections

- Cats and dogs: Up to 400 L_3_ SC per animal to produce microfilaremic lymphatic infections.
- Jirds and other rodents: Up to 150 L_3_ SC to produce microfilaremic lymphatic infections; ~400 L_3_ SC to produce intraperitoneal infections, particularly in male jirds.

**Standard Operating Procedures for Obtaining *B. malayi* Specimens for this Study at Washington University School of Medicine**

All animals at Washington University School of Medicine were handled in accordance with guidelines defined by the Animal Welfare Act (A3381-01), Association for Assessment and Accreditation of Laboratory Care International (AAAALAC), PHS Policy for the Humane Care and Use of Laboratory Animals, the Guide for the Care and Use of Laboratory Animals, and the Division of Comparative Medicine, Washington University School of Medicine. All animal work was approved under WUSM Institutional Animal Care and Use Protocol 20120025”

**Blood feeding techniques (to feed on animals or by membrane feeding) using laboratory reared *Aedes aegypti* for *B. malayi infections***

1.0 Blood meal using infected Microfilaraemic (Mf+ve) gerbils or Membrane feeding method using shipped blood

1.1 Collect approximately 300-400 female *A. aegypti* pupae into small wide mouth plastic cups and place them in chlorine free clean tap water. Distribute pupal cups as one in each cardboard ice cream carton containing a paper sheet at the bottom. In 2-4 days, many pupae should emerge as adult mosquitoes inside these cartons. These cartons should be tightly closed by nylon screen. Continue to feed adult mosquitoes with sugar cubes and 10% sugar solution using cotton balls.

1.2 Approximately 5-6 day-old adult female mosquitoes are used in blood meals.

1.3 Remove food source (sugar cubes and sugar solution) from the mosquito containing cartons and from pupal cup 24 hr. prior to the blood meal.

1.4 **Direct feeding**: *B. malayi* infected gerbils (> 120 days post SC infection with third stage larvae) are screened for circulating Mf by testing 10 ul of tail vein blood samples. Mf counts (~ 50-100/10 ul of blood) should be used for direct feeding. Anesthetize the animals i.p with DCM approved cocktail in saline. Inject cocktail solution in a volume of 0.20-0.25 ml with tuberculin syringes fitted with 20g needle into i.p cavity. This is to prevent animals moving around while allowing mosquitoes feed on their blood. Short term anesthetization is also recommended to increase Mf counts in peripheral blood which will enhance the L3 recovery rate in mosquitoes. Allow two infected, anesthetized gerbils per carton of mosquitoes for feeding. Place gerbils facing their abdomen on the screen to allow blood feeding. Strictly follow DCM protocol for anesthesia, recovery and minimize stress to animals that may be induced during procedures. Record and sign the document on amount of anesthetic used for periodic inventories. Diluted anesthetic samples should be discarded as per the approved DCM/IBC protocol.

1.5 **Membrane feeding**: Preorder heparinized infected blood samples (~10 ml) from FR3 at least a week prior to the procedure and make arrangements for shipping overnight. Obtain Infected cat blood or normal cat blood spiked with *Brugia malayi* (source may vary each time for Mf that may be derived from an infected cat donor or gerbil donor). Store the blood at RT or 37 C until use. Prepare mosquito infection procedure as soon as the blood samples arrive, (usually 20-24 after shipping). Check Mf counts and Mf viability in 10 ul of heparinized blood. Blood containing high Mf counts should be diluted using normal heparinized cat blood and bring Mf counts suitable for infections.

1.6 Always be prepared to use infected Mf positive gerbils instead, as described above. In some shipments from the repository, blood Mf counts were either very low or Mf were inactive. May be Mf lose viability in shipment and storage in blood for 24 hr or more. Continue to feed some mosquitoes with shipped blood and expect to recover few L3s when Mf counts are too low and if at all they get infected.

1.7 Setup membrane feeding apparatus using sausage casing as a membrane. Maintain circulating water and *B malayi* infected blood temperatures at about 37°C in the inner chamber of the apparatus. Starved mosquitoes feed quickly, and majority of them feed on blood in about 30 to 1 hr. Fully engorged and semi-fed mosquitoes leave the membrane inner surface and begin to move to the inner lining and base of the carton, suggesting feeding may be complete.

1.8 After completing infected blood meals, place sugar cubes and water infused cotton on the carton screen. Make sure water infused cotton is always wet by adding drops of water at 2-3 day intervals until 14 days.

1.9 Maintain infected mosquito colonies in their cartons for 14 days at controlled and recommended humidity and temperature in the insectary located in biohazard facility. Label cages after the blood feed to assess L3 recovery rates at 14 days post infection. L3s may be pooled from different cartons. Count total numbers of L3 by individually hand picking them under the microscope and dispense them in required number in smaller petri dishes containing warm RPMI 1640. Wash them 1x with RPMI 1640 prior to gerbil inoculations or repeatedly for molecular experiments.

2.0 Mosquitoes fed on normal blood should be used for egg collection and to continue the mosquito life cycle. Plan to feed mosquitoes with normal blood at least every 2-3 months interval to maintain higher hatch rate. Dried egg pads should be stored safely in tight containers in the insectary.

**Recovery of Infective third stage larvae (L3) of *Brugia malayi* from Infected *A. aegypti***

**L3 recovery and gerbil (*Meriones unguiculatus*) infections:**

***1 Brugia malayi* infected mosquitoes and normal mosquitoes should be maintained in temperature controlled insectary located in BSL2 Biohazard facility in WashU.**

1.1 Remove sugar cubes and water-soaked cotton from the top of screens placed above the ice cream cartons (on the day of L3 recovery)

1.2 The mosquito cartons should be placed in a freezer compartment for approximately 5-8 minutes.

1.3 After all mosquitoes are immobilized, remove screen of the carton. The infected mosquitoes (maximum of 1000 mosquitoes) collected from two or more cartons should be placed on a chilled glass plate and gently crushed in warm RPMI 1640 Medium (no antibiotics supplemented at this step)

1.4 The crushed mosquitoes are then placed on a nylon sieve immersed in warm RPMI 1640 and that was submerged in a conical wine/beer glass. Quickly remove the screen with crushed mosquitoes and place it in another beer glass with RPMI, and allow L3s migrate to the bottom of the wine glass when maintained at 30- 32 C

1.5 The L3 settled at the both of the wine glass are collected twice at 15 to 45 minutes as two collections. Most active and fully grown L3s are migrated out of the mosquitoes and into the bottom of the wine/beer glass during this time.

1.6 L3s are collected into 10mm round flat bottom petri dishes in fresh RPMI 1640 (no antibiotics supplemented) and individually hand- picked with glass micro pippettes into smaller petri dishes containing RPMI. Use dissecting microscope for L3 collections. The L3s are collected in 50, 100, 150 or, 200s based on the experimental protocol. Gerbils are usually infected with 100-150L3s /animal for subcutaneous infections and in 200s for i.p infections.

1.7 L3s are placed in 1 CC tuberculin syringes in less than 0.5 ml warm RPMI 1640 medium and by using 20G needle are njected SC or ip into young male gerbils (6-8 weeks old and supplied by CharlesRiver Labs).

***Brugia malayi* infections in gerbils with mosquito derived infective larvae (L3) and recovery of adult worms, microfilariae for experimental research**

1.0 Place an order of male Mongolian gerbils (*Meriones unguiculatus*) from CharlesRiver Labs approx. 2-3 weeks prior to the inoculation day with L3. Order 4-6 wks old male gerbils. In house breeding for animals is not approved by DCM for this protocol. DCM will purchase the required numbers and house them. Animals are available to use after a brief quarantine at the facility.

1.1. Recover pooled L3, and collect them as described above in 50, 100, 150 or 200 or more as required for the experiments. Collect L3 in small Petri dishes with warm RPMI 1640. Wash them 1X prior to inoculating them into animals. Wash them at least 3X when used in molecular work.

1.2 A dissecting scope and glass pasteur pipettes tapered at the end are used to collect the L3s from small petri dishes. It is helpful to keep petri dishes slightly tilted to allow L3s settle at the bottom.

1.3 Collect settled and pooled L3s in a small volume of the RPMI medium into 1-cc tuberculin syringes and cap them with 20g needles. Prevent needle injuries by capping them with plastic tops. Inoculation should be performed in DCM animal facility within 2-3 hours after recovery.

1.4 For subcutaneous injection collect 100-150 L3s from pooled L3s sample per animal and for intraperitoneal inoculations collect 200-300 L3s / animal). Subcutaneous infections are recommended to inject pooled L3s in the inguinal region of male gerbils and ip infections are usually administered in the abdomen into the peritoneal cavity. Make sure to dispense most of the larvae into the animals.

1.5 Label animal cages with date of infection, route of infection and initials of the person performed the procedure.

1.6 It is recommended to maintain all infected and normal animals in the animal facility by DCM, and experimental protocols are not approved to bring them outside DCM.

1.7 Begin monitor the infection status in s.c infected animals after 120 days post-infection by taking a blood sample from the tail vein and look for circulating Mf/in 10ul blood under compound microscope.

**Recovery of adult worms from i.p infections**

**1. Fully mature adult worms will be ready to collect around 120 days post-infection and later.**

1.1 Prepare warm HBSS medium

1.2 Euthanize i.p animals with *B. malayi* as per the DCM euthanizaiton protocol that was approved

1.3 Infuse 5-10 mls of warm HBSS medium containing heparin into the peritoneal cavity, collect peritoneal lavage containing Mf, adult worms and host material. Mf recovered from i.p are not necessarily at the same stage, it will be a mixed population with some underdeveloped.

1.3 Wash adult worms 1-3x with HBSS, place them in warm RPMI 1640 in a 10mm petridish. Collect male and female worms separately, count the number of worm recoveries, and snap freeze them in dry ice or liquid nitrogen before collecting them into 1.5 ml Eppendorf tubes and freeze them at -80 or ship them out in dry ice.

1.4 Process peritoneal lavage for Mf isolation (see protocol)

1.4 To recover worms from s.c infections, collect body parts-heart, lungs, spermatic lymph vessels and nodes, testes. Tease out these tissues with fine tweezers into 10 mm Petri dishes containing RPMI 1640 at RT or 37C. Use a dissecting scope to find worms, collect and wash them with RPMI prior to drug testing or molecular work.

**Standard Operating Procedures for Obtaining *B. malayi* Specimens for this Study at Liverpool School of Tropical Medicine**

All experiments on animals at Liverpool School of Tropical Medicine were approved by the ethical committees of Liverpool School of Tropical Medicine and the University of Liverpool and were conducted according to Home Office Legislation, the revised Animals (Scientific Procedures) Act of 1986 (project license numbers 3002974, P86866FD9).

***Brugia malayi* life cycle maintenance**

**Hazard Information**

Appropriate personal protective equipment should be worn during blood feeding procedures.

All staff must take the utmost care to prevent the escape of adult mosquitoes from cages or larval trays. Any adult insects that do escape must be eliminated immediately using an appropriate bug swatter.

Ensure that the insectary is maintained as cleanly as possible and clean the work surface prior to and after use with Virkon solution and dry using paper towels.

Dispose of all waste materials quickly and appropriately. Biological waste materials including mosquitoes should be autoclaved.

**Procedure: Egg laying and Aedes maintenance**

All rearing takes place in the insectary -The insectary rooms are maintained at 26 - 28°C and 84 - 88% humidity, with a 12 hour day/night cycle with 30 minutes dusk.

(Day 1 – Usually on a Wednesday) Select cages containing 5 day old adult mosquitoes for blood feeding as follows:

Using the Hemotek blood feeding system, cut a square of parafilm measuring approximately 6 x 6 cm, stretch over the aperture of the meal reservoir and secure using the 'O' ring. Remove any pleats and creases from the edge by carefully pulling the corners. When the correct tension has been attained, trim the surplus parafilm with scissors. Hold the reservoir so that the parafilm is unsupported and fill with uninfected human blood through one of the two ports using a transfer pipette. Next seal the filling plugs with plastic plugs. The capacity of the reservoir is approximately 5 ml.

The feeding area of the parafilm is approximately 10 sq.cm. (sufficient area for 50 female mosquitoes to feed together). Attach the prepared reservoir to the feeder by screwing it onto the stud on the heat transfer plate at the bottom of the feeder. Then plug the feeder into the power unit and place the feeder on the insect cage. The feeder has been designed so that it will stand on top of the cage to enable the insects to feed through the mesh of the cage.

Allow the mosquitoes to feed for at least 90 minutes but remove the feeder and clean it before the end of the day.

The females will lay eggs two days after they feed on blood. Place a piece of cotton wool pad into a pupae pot containing distilled water, making sure that the cotton wool is moist but that no free water is available for mosquitoes to drown themselves in.

This is then kept inside the cage for the mosquitoes to lay eggs; leave the egg pot in the cage for 7 days. Note that eggs from *Aedes* mosquitoes may be dried on the cotton wool and stored in a sealed container at room temperature for several months. Eggs stored in this way will lose about 10% viability per month. Eggs are dried before hatching and are left in insectary for the drying process.

**Day 1.**

When the cotton wool is dry the mosquito eggs are placed into a larval tray with tap water and the water topped up to about 2cm depth. Add a half of a yeast tablet to the larval tray. Place the tray on a rack and do not move the tray - keep it perfectly still overnight.

**Day 2.**

Just observe the eggs making sure they are hatching from the cotton wool pad do not add any more yeast and do not move tray.

**Day 3.**

When the eggs have completed hatching, the larvae should be split into as many larval trays as required using tap water and again filled to a depth of about 2cm. Add a half of a yeast tablet to the larval tray.

Monitor the tray closely for density and population, keep splitting the larvae with tap water into further larval trays until the density of the larvae is around 250 per tray. You may obtain up to about 15 trays of larvae from a single egg paper. *Aedes* mosquitoes grow in dirtier water so cleaning is not as important.

**Day 4 – 6.**

Just leave the larvae to grow in the trays from stages L1 – L4, do not add more water if it becomes dirty and do not add more yeast tablets. This will just make the water stagnant and kill the larvae due to lack of oxygen in the water.

**Day 7.**

On this day you will notice pupae will be developing and the L4 larvae will be diminishing just leave for another day for all larvae to turn to pupae.

**Day 8.**

As we have mass reared the pupae the pupae may be removed by filtration with an appropriate gauge fish or other net. Pour 4 trays of pupae into the net then place the collected pupae into a pupae pot with about 1.5cm of cold water. Place the pot into a bugdorm-1 cage and place a wet sugar coated cotton wool pad on top of the cage to provide nutrients and leave for 5 days for all the pupae to emerge. Do not feed pupae once placed in the adult cage up to about 1,000 adults can be reared in a single cage.

The adults are then kept in the insectary room for 8 days. Feed the adults using 10% sucrose solution by placing a cotton wool pad on the top of the cage each day. The cotton wool pad should be wet with 10% sucrose solution but not dripping liquid and should be changed daily.

Adult mosquitoes are normally used after 8 days post emergence, sometimes they will be utilised up to about day 10. The adults will continue to live for up to about 1 month after hatching if allowed to.

Mating occurs at dusk or in a darkened room or cage. Maximum mating occurs 3-5 days after emergence. Fertilized females take a blood meal more readily than unfertilized. If the mosquitoes are to be blood fed then the adults (both males and females) are kept in the insectary room for 4-5 days, fed on 10% sucrose before they are again blood-fed to begin the next cycle. The same mosquitoes can be used to lay eggs more than once.

All larval trays, pupae pots and adult cages must be cleaned as soon as possible prior to using again using 2% Decon 90 or other appropriate laboratory cleaner.

**Procedure: Filarial nematode maintenance in animals**

Filarial nematodes (*Brugia malayi* and *Brugia pahangi*) are maintained in the peritoneal cavity of jirds (Mongolian gerbils) or mice. These are either implanted (adult parasites) or injected (L3 larvae) directly into the peritoneum in the Biomedical service unit (University of Liverpool).

Adult parasites can be retrieved from the rodent host to conduct *in vitro* experiments or for analysis following *in vivo* drug treatment. Microfilariae can be retrieved to conduct *in vitro* experiments, for analysis following in *vivo drug* treatment and also to maintain the life cycle.

THIS WORK SHOULD BE CONDUCTED IN CTID ANIMAL HANDLING ROOM CT026. If the biosafety cabinet in this room is unavailable or the room itself is unavailable then the procedure can be conducted in CT245, using biosafety cabinet SCR-303. Please refer to SOP FIL-SOP-CUL-017 for the transport of the infected animal to this lab.

**Microfilarial infection in *Aedes***

*Brugia malayi/pahangi*: Infected gerbils housed at the Biomedical services unit (UoL) undergo peritoneal lavage (tapping) to retrieve either *Brugia malayi* or *Brugia pahangi* microfilaria. Microfilaria is then transported back to CT240, CTID building, LSTM, in a sealed storage container. Once the microfilaria have been obtained from the gerbil peritoneal cavity they need to be counted to establish the amount of microfilaria present. This is done by making a dilution of the mf present by adding 990µl of warm RPMI media containing 5mls of Penicillin-Streptomycin to a 1.5ml Eppendorf tube and adding 10µl of the microfilaria; do this in three separate eppendorfs. Then give all tubes a gentle mix and from each tube take 10µl and spread this along a glass slide from left to right.

Place the slide under a light microscope at x4 magnification and go to one end of the line and count along and record number of mf; do this for all three samples. Take the average of the counts and this will give you the amount of mf per ml. The concentration of microfilaria that is used for the feeds is 20,000mf/ml of blood (this has been found to be the best concentration to use for feeding the mosquitoes). Once calculated place the number of mf needed into 40mls of blood and this will enable you to feed 15 cages to get a good infection. Leave the cages for 14 days after feeding to establish the infection in the insectary, changing the sugar pads daily.

**Obtaining the L3 from the mosquitoes**

The cages of infected mosquitoes are carried in a sealed plastic box big enough to contain two mosquito cages. Up to 15 cages of mosquitoes are used for infected feeds. They are carried from old school insectaries to the CTID building room CT240 on a trolley via stairs and lifts.

The mosquitoes are then placed in a cold room at 4°C in CT222 to chill down before crushing for approximately 20 minutes.

RPMI 1640 medium is warmed in a water bath at 37°C for approximately 30 minutes and 5ml of Penicillin-Streptomycin is added to the medium.

The crushing of the mosquitoes is carried out in CT222, once chilled down the mosquitoes are placed on a glass plate 300 x 300mm with approximately 3mls of warm RPMI 1640 medium and a 25ml pipette is used in a rolling pin motion to crush the mosquitoes. The crushed mosquitoes are then washed off with approximately 5-10mls of the RPMI into a glass beaker using a 5ml plastic Pasteur pipette.

A retort stand is set up with two clamps to hold the glass funnel in place, the sieve is placed into the neck of the funnel and approximately 400ml of the warm RPMI media is poured in through the sieve and the rest is used for the crush. A plastic Pasteur pipette is then used to remove any air bubbles from the underneath of the sieve if this is not done any L3 larvae will not get through the fine pores on the sieve and transported down the funnel. The media with the crush in is then poured through the fine 70µm sieve and left for 90 minutes. A clip is then used to tie the clear rubber hose approximately 4 inches from the bottom, this media will contain all the L3 and stop excess media from being taken into the petri dish. The resulting L3 are then decanted off into a petri dish and are counted via a microscope. The L3 are removed using a 20ml Gilson pipette with a 200µl pipette tip attached.

The required number of L3 are counted out into wells on a 96 well Plate and then taken up using a 1ml syringe and a 25G 5/8” needle at 50 - 200 larvae per syringe. The syringe is placed into a secure plastic box and then transported over to the animal house with heat pads at 37°C where we inject the animal of purpose i.e gerbil or mouse to continue the life cycle.

Once the gerbil is infected with L3 it will be left for three months for the microfilaria to develop and a tap to be performed to start the infection of the mosquito once again.

All plastic pipettes, plastics, glassware and sieve are soaked in 1% virkon then washed. The crush area is cleaned with 1% virkon.

**Standard Operating Procedures for Obtaining *B. malayi* Specimens for this Study in Lucknow, India**

The study bears IAEC approval number 129/08/Para/IAEC/renew (84/09) dated 27. 04. 2009.

**Experimental Maintenance of *Brugia malayi* Infection**

**Vector**

Rearing and breeding of mosquito vector Aedes aegypti is carried out in temperature and humidity controlled insectarium (27±1°C temperature, 75±5% relative humidity). Filters carrying dry stored eggs when placed in water bowls hatched into first stage larvae and a fine powder of Yeast tablets and dog biscuit (3:1) was used as a feed for growing larvae. Within 5/6 days, the larvae got transformed into pupae, the non-feeding stage which were recovered with the help of glass pipettes and transferred to water in small crystallizing dishes inside the nylon netted mosquito cages. The young mosquitoes emerging from the pupae were provided with 3% glucose solution in cotton covered on top with moist filter paper disk in small petridishes kept inside each mosquito cage. A small beaker containing water was also placed in the cage for female mosquitoes to lay eggs.

**Host**

*Sub-periodic Brugia malayi* was experimentally maintained in two rodent species, i) *Mastomys coucha*, the red eyed rodent with straw coloured fur originally brought from Giessen, Germany through the courtesy of Late Prof. G. Lammler in 1974 and, ii) *Meriones unguiculatus* (Mongolian jird/gerbil) having Agouti or mixed brown colour fur. The rodents are being maintained in the Laboratory Animal Facility of CSIR-CDRI, Lucknow, India. Breeding of the two rodent species was carried out under proper hygienic conditions and controlled temperature (26±2°C), drinking water is made available ad libitum and animals are fed on a diet initially developed for syrian hamsters (Schuster et al. 1973), supplemented with proteins and fats.

Young (4-5 day old) mosquitoes were fed on the blood of infected donor mastomys between 12.00 and 12.45 h (time of peak microfilaraemia). Mosquito eggs are collected from water surface and filter paper discs, washed well, left to dry completely and stored in desiccators to be used within 6 months. On day eight, 4–5 female mosquitoes are dissected to have an idea of the development of larvae and to ascertain the day of collection of infective third stage larvae (L3). The mosquitoes are crushed gently in sterile Ringer's solution, quickly poured on to the funnel of Baerman's apparatus. Within 40 min, *B. malayi* L3 are collected in small volume of Ringer in glass cavity block, washed repeatedly and mature actively moving L3 were counted and distributed in various cavity blocks. Six week old naive male mastomys were inoculated subcutaneously in the back region with 100 L3 each, while 8-10 week old male gerbils were infected by intraperitoneal inoculation with 150- 200 L3.

Ten microlitre of blood was drawn after needle pricking of the tail tip of mastomys at the time of peak microfilaremia on day 90 post infection and thereafter every month. Thick blood smears are made on glass slide, air dried overnight, dehaemoglobinized and stained with Leishman. Microfilariae are counted in each smear to assess microfilarial densities and donor animals (80–150 mf/10 µl) are selected to infect mosquitoes for cyclical transmission of *B. malayi.* Infected gerbils are used for recovery of adult parasites and microfilariae to be used in various experimental studies including drug discovery.

Adult parasites and microfilariae are recovered by peritoneal washings of jird after 120–180 days of infection. Microfilariae are collected after passing the peritoneal lavage through 5.0 µm membrane filter. Adult worms in the case of mastomys are recovered from heart, lungs, testes and peripheral lymph nodes.

**Standard Operating Procedures for Obtaining *B. malayi* Specimens for this Study at Chiang Mai University**

The protocol for samples obtained from Thailand was approved by the Institutional Animal Care and Use Committee (Protocol Number 15/2562) of the Faculty of Medicine, Chiang Mai University, Chiang Mai province, Thailand.

***Brugia malayi* life cycle maintenance**

- **Mosquito: autogenous *Tanakaius togoi***

This laboratory colony-strain of *Ta*. *togoi* exhibited autogenous behavior, thus, the autogenous colony was established by pooling first egg batches of nonblood-feeding adult females from a stock colony. The hatched larvae were reared to the next generation; nonblood feeding adult females were allowed to oviposit eggs again, and these processes were repeated continuously to establish the autogenous colony. The rearing of autogenous *Ta*. *togoi* colony take places in insectary room at 27±2 °C and 70–80% relative humidity, with fluorescent lighting for approximately 12 hr per day.

1. The filter paper with dry eggs was placed into the bowl contained with tap water for hatching.

2. The first stage larvae (L1) were hatched within 24-48 hr after immersion and then transferred to a new rearing tray.

3. Approximately 300L1 were reared in a plastic tray (25 x 35 x 6 cm) containing 2,000-3,000 ml of tap water.

4. The 0.5 g of fine dog biscuit suspended in 10 ml of tap water was added every 3 days for L1 and L2, and this schedule was increased to every other day using 1.0 g of fine dog biscuit suspended in 10 ml of tap water after most of the larvae had reached L3 and L4.

5. To avoid scum forming and/or rotting in rearing water, due to unconsumed food and larval waste products, the water was changed twice weekly or immediately whenever signs of scum and/or rotting is observed.

6. After pupation, the pupae were removed from the rearing tray and transferred to plastic cups containing distilled water. With about 300 pupae, the cups were placed into netted cages (30 x 30 x 30 cm).

7. After emerging, adults were fed with 10% sucrose and 10% v/v multivitamin syrup solution, and mating occurs 3-5 days after emergence.

8. The eggs were laid on filter paper soaked with water in a plastic cup. They were kept for 3-4 days to air-dry before use in the next cycle. The deposited eggs on filter paper and kept in a plastic bag could be stored for at least 6 months in a refrigerator.

- **Infection of *Ta*. *togoi* with nocturnally subperiodic (NSP) *B*. *malayi* microfilariae (mf)**

1. The male Mongolian jird (*Meriones unguiculatus*) inoculated intraperitoneally with NSP *B*. *malayi* L3 for at least 5 months was anesthetized deeply with ethylene ether.

2. The microfilariae were collected by injecting 3 ml of Hank’s balanced salt solution (HBSS; pH 7.2–7.4) into the peritoneal cavity using a 5 ml syringe and 21G x 1" (0.8 x 25 mm) needle, while slightly shaking the abdomen so that the mf mixed well with the HBSS before withdrawing the HBSS-*B*. *malayi* mf mixture with the same instruments.

3. The 0.05 ml of HBSS-*B*. *malayi* mf was mixed first with 5 ml of human-heparinized blood taken from principal investigator or bovine/swine-heparinized blood (10 units of heparin/ml of blood).

4. Microfilarial density was adjusted to approximately 200-350 microfilariae (mf)/20 µl before feeding mosquitoes.

5. After the withdrawal of HBSS and mf, the abdominal cavity of the jird was exposed and rinsed twice with HBSS to recover adult worms.

6.  Five-day-old adult female *Ta*. *togoi* fasted for 24 hr and then were allowed artificial membrane feeding on blood containing *B*. *malayi* mf.

7. Fourteen days after feeding, all infected mosquitoes were dissected in 0.85% normal saline solution and examined under a dissecting microscope. The number of L3s per infected mosquito ranged from 1-40 larvae.

8. A total of 120 harvested L3s from infected mosquitoes were suspended in 0.5 ml of HBSS. Then, they were inoculated intraperitoneally into the right abdomen of a male jird.
